# Versatility and Benefits of 4.0mm Flexible Nasal Endoscopy in 118 Children up to 10 Years of Age

**DOI:** 10.7759/cureus.22656

**Published:** 2022-02-27

**Authors:** Ved P Narang, Anna Loroch, Giovanni Sambiagio

**Affiliations:** 1 Otolaryngology - Head and Neck Surgery, University Hospital Monklands, Airdrie, GBR; 2 Ear Nose and Throat (ENT), NHS Greater Glasgow and Clyde (GGC), Glasgow, GBR

**Keywords:** 4.0 mm, otitis media with effusion, local anaesthesia, endoscopy, adenoids

## Abstract

Purpose

This retrospective study looked at the feasibility of using adult 4.0 mm flexible nasendoscopes (FNE) examination under local anesthetic (LA) in children three to 10 years old to diagnose adenoid hypertrophy (AH) and other conditions. We also looked for a correlation between the adenoid size on FNE and a) tonsil size, b) the typical symptoms of snoring, mouth breathing, impaired hearing, and apnoeic episodes c) the management options of otitis media with effusion (OME) and d) the adenoid size intraoperatively.

Methods

A retrospective, observational study of 118 children in an NHS pediatric otolaryngology clinic led by a single consultant. One hundred ten consecutive patients with suspected AH were divided into two groups of three to five years and six to 10 years. We compared the acceptance rate to FNE in two subgroups (three to five years and six to 10 years old) and examined the correlation between various parameters as outlined above, using the Chi-square test. Eight children underwent FNE for other reasons of change of voice and epistaxis.

Results

FNE was successfully performed in 86% of the patients without restraint. Thirty-three percent of patients had non-obstructive adenoids (OA) and did not require surgical intervention. The intraoperative adenoid size, symptoms of snoring, mouth-breathing, and apnoeic episodes positively correlated with OA; however, no correlation was seen with the tonsil size (p=0.1143). All patients with OA and type B tympanogram needed adenoidectomy and grommet insertion (p=0.0119), and those with type C curves recovered with adenoidectomy alone.

Conclusions

4.0 mm adult scope helped reach a definitive diagnosis for AH in most children above three years of age, thus proving cost-effective. The symptoms of snoring, mouth-breathing, and apnoeic episodes had a positive correlation to the presence of OA; however, the tonsil size was seen to be independent of adenoid size.

Primary surgical management can be considered the treatment of choice for all patients with OA and type B tympanogram without a trial of conservative therapy.

## Introduction

Snoring, mouth breathing, and apnoeic episodes are a source of great distress and anxiety for many parents. Parents often express grave concerns, record videos, and commonly feel the need to quiver their child to stimulate breathing when confronted with an apnoeic spell.

Although there are many reasons for a young child to develop symptoms of snoring, mouth breathing, apnoeic episodes, and nasal discharge, enlarged adenoids by far remain the most common cause [1,2]. The diagnosis is often based on a parents' account of symptoms and clinical examination that does not involve assessing the post-nasal space. It has not been widespread practice across the UK to offer flexible nasendoscopy (FNE) for the pediatric population of patients. One of the reasons might be suspicion of non-cooperation and poor tolerance of the procedure under local anesthesia in the outpatient setting [3].

Aims

This study aimed to assess the feasibility of using 4.0 mm flexible nasendoscopy in the three to 10 years age population as a diagnostic tool in evaluating adenoid hypertrophy and other diseases. We also wanted to assess the correlation between adenoid hypertrophy (AH) and common adenoidal symptoms of snoring (S), mouth breathing (MB), impaired hearing (IH), nasal discharge, and apnoeic episodes (AE). The FNE findings also compared the intraoperative adenoid size, the clinic tonsil size, and the management options of otitis media with effusion (OME).

The article's findings were presented at the 28th European Rhinology Society Congress on September 28th, 2021" in Thessaloniki, Greece.

## Materials and methods

Data reported in this study was derived from a retrospective observational study of 118 consecutive patients presenting to the pediatric otolaryngology clinic with common adenoidal symptoms and other symptoms of change of voice, grunting, epistaxis, and anosmia. They were offered FNE in an outpatient clinic led by a single consultant.

For the analysis, 110 children with suspected AH were divided into subgroups of three to five years and six to 10 years of age. The age limits were set on the premise that children above 10 years are likely to cooperate with the endoscopic assessment, whereas assessing patients younger than three years old might be too distressing for the child and the parents; hence we excluded them from the study group. We also excluded children with craniofacial deformities, learning disabilities, syndromic children, and eight children who underwent FNE for other symptoms.

All patients meeting guidelines for tonsillectomy could undergo an examination under general anesthesia (GA) for AH. FNE was avoided [4] in such patients until insisted by the parents, and they were excluded from the study group.

The patient sat independently upright on a chair without restraint, with the parent by their side. One puff of local anesthetic (LA), i.e., 5% Xylocaine spray, numbed the wider nasal cavity and facilitated the passage of FNE with a mounted camera head. The examining clinician followed the image on the screen of a stack system. A screenshot captured the adenoid size using the switch control on the camera head.

We used Cassano's classification [5] to grade the size of adenoids from 1 to 4, depending upon the percentage of posterior choanal space blocked by the adenoids. It was categorized as grade 1 for less than 25% blockage to grade 4 for more than 75%, in intervals of 25% each [5,6]. Grade 1 and 2 adenoid sizes were classified as non-obstructive (non-OA) and grade 3 and 4 as obstructive adenoids (OA).

Brodsky's classification [7] allowed us to grade tonsil size from 1 to 4 by estimating the width of the oropharynx occupied by the tonsils. Grade 1 tonsils occupied less than 25% of the oropharyngeal space, gradually increasing in intervals of 25% each to reach grade 4 when occupying more than 75% of the oropharyngeal area. The presence of OME was diagnosed with a history and clinical examination (including pneumatic otoscopy, audiometry, and tympanometry).

The surgery was carried out under GA by the lead author and his colleagues in the department, as this surgery is listed in a shared pool. The audit team accessed the surgical notes to retrieve the size of the adenoids observed in the operating room (OR) and the procedure performed for OME.

Statistical methods for outcome measures

The Chi-square test using the stats library in the SciPy package for Python 3.0 (www.python.org) assisted in completing the data analysis. Mean, median, and standard deviation describe the continuous data, and the percentages represent the category variables. A p-value of <0.05 is considered statistically significant.

## Results

One hundred ten patients underwent FNE under LA for AH. The mean age of the patients was 5.2 years (standard deviation [SD] 1.8). The minimum age of the children included in this study was three years and one month, and the maximum was 10 years.

The study group was split into two subgroups of three- to five-year-old patients (49 children, representing 45% of all patients, with the mean age of 4.4 years) and six- to 10-year-old patients (comprising 61 children, 55% of all patients, with the mean age of 7.6 years). 87.27% (96) children were between three and eight years old, and the largest group of patients was at the age of four to five years (27 children).

Eight children refused to cooperate in the three- to five-year subgroup, and the same number declined in the six- to 10-year subgroup. The chi-squared test showed no statistically significant difference existed in accepting the FNE between the subgroups (p=0.8934) (Table 1).

**Table 1:** Number of patients accepting and refusing FNE in various age groups. p-value = 0.8393 – Insignificant, when comparing the two groups. FNE: flexible nasendoscope

Age group	Average age in months (Years)	Total Number	Number refusing FNE	% Patients refusing FNE under LA
3 to 5	53.48 (4 years 5 months)	49	8	16.32
6 to 10	91.08 (7 years 7 months)	61	8	13.11
All children under 10 years	74.61 (6 years 3 months)	110	16	14.54

Seventy-seven patients (70%) presented with snoring and mouth breathing, 14 (12.72%) had a history of apnoeic episodes and 20 (18%) presented with associated complaints of impaired hearing.

A positive correlation was found between OA and symptoms of snoring (p=0.0224), snoring and mouth breathing (p=0.0076), and apnoeic episodes (0.0088). Such correlation was missing in patients presenting with impaired hearing (p=0.6712) (Table 2).

**Table 2:** Symptoms and corresponding sizes of adenoids on FNE *The total of this column is more than 110, as patients could have one or more than one subgroup of symptoms. p-value for snoring & mouth breathing = 0.0001 - Significant. p-values for snoring & apnoeic episodes = 0.0224 & 0.0088 – Significant p-value for impaired hearing = 0.6712 - Insignificant FNE: flexible nasendoscope

Symptoms	Grade 1	Grade 2	Grade 3	Grade 4	FNE Refused	*Patients with symptoms	P Value
Snoring (S)	3	0	3	1	0	7	0.0224
Snoring + Mouth breathing (S+M)	2	18	28	16	13	77 (70%)	0.0076
Recurrent Nasal discharge (ND)	2	1	0	0	0	3	0.0107
All three symptoms of S, M & ND	1	3	4	5	1	14 (12.72%)	0.5790
Impaired hearing	3	3	8	4	2	20 (18.18 %)	0.6712
Apnoeic Episodes	0	3	11	0	0	14 (12.72%)	0.0088

The adenoid size on FNE, when compared with the size of tonsils at the time of clinical examination, was seen independent of each other (p=0.1143) (Table 3).

**Table 3:** Adenoid size on FNE vs tonsil size at time of initial clinic examination p-value = 0.1143 – Insignificant. FNE: flexible nasendoscope

Adenoid Size	Non-Obstructive Adenoids	Obstructive Adenoids
Tonsil Size		
Grade 1	8	1
Grade 2	14	10
Grade 3	30	4
Grade 4	12	9

Sixty patients were diagnosed with OA on FNE. At the time of writing this paper, 42 patients underwent surgery. Twelve (29%) had moderate and 29 (71%) had large adenoids, according to the documented intra-operative findings. The size was not mentioned in one patient. The adenoids were removed in all patients listed for the surgery.

Four patients did not have surgery in our unit due to either behavioral issues, referral to the tertiary center due to comorbidities, or parents' refusal to proceed with the surgery. One was lost to follow-up.

An additional eight patients had their adenoids removed. Five patients had refused FNE, and three with non-OA had their adenoids removed whilst undergoing tonsillectomy. Out of 16 patients refusing FNE, five underwent surgery, and six continue to wait for their procedure. Three patients from this group reported improvement; hence they were discharged from the clinic, and two were lost to follow-up (Table 4).

**Table 4:** Adenoid size on FNE vs adenoid size in the operative room @ Four patients with OA did not undergo surgery due to other reasons (see text) * Five patients refusing FNE were discharged as parents reported improvement in three of them, and two failed to follow up. $ Three patients with non-OA had their adenoids scraped along with tonsillectomy. 42 patients with OA by FNE underwent surgery, and all had their adenoids removed. 12 (29%) had moderate, 29 (71%) had large with no mention of adenoid size in one patient. FNE: flexible nasendoscope, OA: obstructive adenoids

Grade of adenoids	Number of patients	Patients D/S after FNE	Patients waiting for surgery	Patients Undergoing Surgery	Non -OA Small	OA - Moderate + Large	Size Not mentioned
Non-Obstructive Adenoids	34	31	0	3	^$^3	0	0
Obstructive Adenoids	60	^@^4	14	42	0	41	1
Refused	16	*5	6	5	0	5	0
Total	110	40	20	50	0	49	1

Of the 94 patients undergoing FNE, 31 (33%) did not require surgery as they had non-OA and were discharged from the service. Four were discharged for other reasons, as explained under Table 4. A total of 50 patients had their adenoids removed, and 20 are awaiting surgery (Table 5).

**Table 5:** Final Analysis of all patients who were offered FNE 31 (33%) with non-obstructive adenoids were discharged to home as they did not surgery. *9 patients were discharged for other reasons FNE: flexible nasendoscope

Total Patients	110
Total patients accepting FNE	94
Patients Refusing FNE	16
Patients discharged after FNE (Non-Obstructive Adenoids)	31
*Patients discharged because of other reasons	9
Received Surgical Intervention	50
Awaiting surgery	20

Out of 20 patients presenting with additional complaints of impaired hearing, five with non-OA had complete recovery of hearing with conservative management. All nine patients with OA and type B tympanogram proceeded to have adenoidectomy/adenotonsillectomy and insertion of grommets (p=0.0119). Two patients with OA and type C tympanogram recovered with adenoidectomy alone. Three patients are awaiting surgery, and one was lost to follow-up (Table 6).

**Table 6:** Adenoid vs OME Given that the 16 patients recovered from impaired hearing, all 5 with grade 1 or 2 adenoids recovered without surgery. In comparison, 9 of 11 grade 3 or 4 FNE patients required tympanostomy tube insertion; we find the p-value at 0.0119, which allows us to conclude a statistically significant positive correlation between larger adenoid sizes in FNE, presence of OME, and requirement for tympanostomy tube insertion. FNE: flexible nasendoscope, OA: obstructive adenoids, OME: otitis media with effusion

Number of patients with Impaired Hearing + adenoid hypertrophy	20
Adenoidectomy (OA) + tympanostomy tube insertion or *Adenotonsillectomy + tympanostomy tube (Type B Curve)	9
Only adenoidectomy (Type C Curve)	2
Waiting for surgery (2 with OA, 1 with non OA)	3
Patients' hearing improved without surgery (Non OA)	5
Lost to FU	1

## Discussion

The stress of subjecting a child to a procedure under GA without an established diagnosis can cause significant apprehension in parents. A myriad of techniques has been attempted in the past to confirm the diagnosis of AH. These include postnasal mirror examination, radiography of the nasopharynx, Magnetic Resonance Imaging (MRI), acoustic rhinometry, rigid endoscopy, ultrasound, or an examination under anesthesia (EUA), to name a few [8].

Flexible nasal endoscopy under LA is safe, easy to perform, and a well-known technique for assessing the size of the adenoids [2,5,8]. The past studies have used small caliber scopes [9]; however, we did not experience any difficulty in using standard 4.0 mm scopes. The 4.0 mm size is readily available in adult clinics; however, we found limited evidence in the literature of their use in the pediatric age group under LA. Thus, the purchase of a narrow diameter scope can be set aside, making the 4.0 mm scope more cost-effective. The use of small caliber scope may be helpful in some children with narrow nasal passages [2,4,9]; however, they have reduced image resolution [10].

The collapse of the soft palate in a supine position may impact the surgeon's ability to evaluate the adenoid size correctly [3]; hence, all patients underwent an examination in a sitting position. Although previous studies have reported using mild physical controls to perform the FNE [9], we did not use any restraints. Eighty-six percent of our patients successfully underwent FNE, while 14% failed to cooperate. A formal record of the reasons for the failure of children to cooperate was not maintained; however, the single consultant who performed all the endoscopies could recall apprehension in children and intense nasal irritation to 5% Xylocaine nasal spray to be the most common causes. The refusal rate is statistically insignificant (p>0.893) between the two groups, concluding that age is not the limiting criterion. In our experience, thoughtful and considered counseling for both the child and the parent is paramount in determining the success of the procedure.

The common presenting symptoms underwent evaluation for their correlation with OA. Snoring (p=0.0224), "snoring and mouth breathing" (p-value 0.0076), and apnoeic episodes (p=0.0088) had a significant correlation with OA; however, the presence of impaired hearing (p=0.6712) is insignificant. The presence of S, S+MB, and AE can be used as a solid indicator to suspect OA in patients refusing FNE.

The lateral X-ray of the nasopharynx could be a cost-effective tool [11], but they are no longer considered reliable [12]. They have also been criticized for depicting a three-dimensional, dynamic space as a two-dimensional static one [13] and for the harmful effects of radiation. We did not subject any of our patients to X-rays. The authors feel they are unnecessary as FNE offers an immediate diagnosis without the need for potentially harmful investigations.

A screenshot of posterior choanae allowed us to counsel parents on adenoid size according to Cassano classification [5] instead of verbally explaining anatomical structures to them [14]. In our experience, we found a screenshot to be a simple and effective tool to explain the pathology and proposed management to the families.

Thirty-three percent of patients with non-OA on FNE were sent home without surgical intervention; their parents explained that the child's symptoms were unrelated to the adenoid size, and we would look for an alternative diagnosis. Despite the negative findings, these children benefited from this procedure in the outpatient setting by avoiding an unnecessary examination under GA. We suggest that this would lead to substantial cost savings and release bed capacity for healthcare providers.

Twenty-nine percent of patients who underwent surgery due to OA had a moderately sized adenoid, and 71% had large adenoids when examined intraoperatively. Since all patients scheduled for adenoidectomy were confirmed to have OA on examination under GA, and no discrepancies were noted in size between the outpatient and intraoperative examination, it validates the effectiveness of FNE for adenoid assessment in an outpatient setting.

Another eight patients underwent adenoidectomy during the same period, out of which five had refused FNE, and three had non-OA on FNE. In three patients with non-OA, the surgeon preferred to remove them at the time of tonsillectomy using mirror examination to assess the size of adenoids. Mirror examination in the OR is not the most accurate method [6] for adenoid assessment. The discrepancy in size between FNE and intraoperative assessment could be due to the recent inflammatory process (e.g., upper respiratory tract infection [URTI]) [3] prompting the surgeon to remove them.

Adeno-tonsillar hypertrophy is a common term used in ENT practice, and it is not unusual to think that the size of adenoids and tonsils can correlate. A large study of 991 children presenting with upper airway obstruction found this correlation to be statistically significant (p<0.0001) [15]. However, in our cohort of patients, the sizes appear independent of each other (p=0.1143). Our study has limitations in reflecting data of patients presenting predominantly with symptoms suggestive of adenoid hypertrophy only.

Twenty patients had additional complaints of impaired hearing at the time of presentation, out of which five had non-OA, and 15 had OA. All five patients with non-OA had their hearing return to baseline at the three-month follow-up appointment by conservative management [16]. Careful observation and conservative management in this group of patients could be considered a treatment option.

Of the 15 patients with OA, 11 patients underwent surgery and four are awaiting their operation when writing this article. Two patients over four years of age had OA and a type C tympanogram. They underwent adenoidectomy only and had improvement of their ear symptoms. Thus, simple adenoidectomy for symptoms of OA also improves Eustachian tube dysfunction and can be considered in these patients. Adenoidectomy alone can hasten the resolution process of OME and decrease the use of grommets [17-19]. However, it contrasts with the present recommendations where a combination of adenoidectomy and grommet insertion finds favor in children over four years of age [16,20].

The choice of placing a grommet or a simple myringotomy may vary, depending upon surgical findings and the surgeons' preference. Simple myringotomy is associated with fewer complications such as otorrhea and alteration of tympanic membrane structure [21]. Grommets, however, can have more reliable short-term results and an inherent advantage of prolonged middle ear ventilation [21,22]. Nine patients with OA and persistent type B tympanogram required adenoidectomy and grommet insertion as an outcome. Initial conservative management with watch-and-wait strategy and auto insufflation [16,20,22,23] failed to improve these patients.

Given that the 16 patients recovered from impaired hearing, five with non-OA recovered without any surgical intervention; whereas nine of 11 patients with OA and impaired hearing required adenoidectomy and grommet insertion as an outcome; a statistically significant positive correlation was noted between the OA, OME and the need for surgical intervention (p-value at 0.0119).

Since all nine patients with OA and type B tympanogram needed adenoidectomy and grommets insertion as an outcome, we contemplate that a proactive surgical intervention without a conservative trial of three months may help them with early recovery and better quality of life. A longer duration of OME decreases the chance of a complete resolution and increase the incidence of retraction pocket, ossicular erosion, adhesive atelectasis, and relapse, thus supporting our suggestion of early surgical intervention [20,22].

Our results are consistent with previous studies, where patients with OA needed grommet insertion as an outcome (p<0.0001) [8], and adenoidectomy enhanced their clinical effectiveness [17]. However, different criteria may apply in children with risk factors for developmental difficulties [23].

## Conclusions

FNE with a 4.0 mm Flexi scope can be inbuilt as a standard and well-tolerated procedure in an outpatient clinic for children over three years of age without the need for additional narrow scopes. It allows us to reach a definitive diagnosis in most children presenting with symptoms suggestive of AH under topical anesthesia and reduces the need for examination under GA.

Since all patients with OA on FNE and a persistent type B tympanogram benefited from adenoidectomy and grommet insertions, it might be worth contemplating a proactive surgical intervention as the primary treatment of choice for such patients.
